# Effects of weak ties on epidemic predictability on community networks

**DOI:** 10.1063/1.4767955

**Published:** 2012-11-26

**Authors:** Panpan Shu, Ming Tang, Kai Gong, Ying Liu

**Affiliations:** Web Sciences Center, University of Electronic Science and Technology of China, Chengdu 610054, People's Republic of China

## Abstract

Weak ties play a significant role in the structures and the dynamics of community networks. Based on the contact process, we study numerically how weak ties influence the predictability of epidemic dynamics. We first investigate the effects of the degree of bridge nodes on the variabilities of both the arrival time and the prevalence of disease, and find out that the bridge node with a small degree can enhance the predictability of epidemic spreading. Once weak ties are settled, the variability of the prevalence will display a complete opposite trend to that of the arrival time, as the distance from the initial seed to the bridge node or the degree of the initial seed increases. More specifically, the further distance and the larger degree of the initial seed can induce the better predictability of the arrival time and the worse predictability of the prevalence. Moreover, we discuss the effects of the number of weak ties on the epidemic variability. As the community strength becomes very strong, which is caused by the decrease of the number of weak ties, the epidemic variability will change dramatically. Compared with the case of the hub seed and the random seed, the bridge seed can result in the worst predictability of the arrival time and the best predictability of the prevalence.

In community networks, the links that connect pairs of nodes belonging to different communities are defined as weak ties. The weak ties hypothesis, which is first proposed by Granovetter, is a central concept in the social network analysis. Weak ties not only play a role in effecting social cohesion but also are helpful for stabilizing complex systems under most conditions. Most recent research results showed that weak ties have significant impacts on spreading dynamics. But until now, no study on the effects of weak ties on the predictability of epidemic dynamics has been given to us. In this study, we investigate how the degree of bridge nodes and the number of weak ties influence the predictability of the epidemic dynamics on a local community. We show numerically that both the degree of bridge nodes and the network modularity are crucial in the predictability of the epidemic spreading on the local community. More importantly, we find out that the variability of the arrival time always displays a complete opposite trend to that of the prevalence, which implies that it is impossible to predict the epidemic patterns in the early stage of outbreaks accurately. This work provides us further understanding and new perspective in the effect of weak ties on epidemic spreading.

## I. INTRODUCTION

Community structures at mesoscale level are ubiquitous in a variety of real-world complex systems,^1^ such as Facebook,^2^ YouTube,^3^ and Xiaonei.^4^ In general, there are more connections between members in the same community than that between members from different communities, where the links that connect pairs of nodes belonging to different communities are defined as *weak ties.*^5–7^ The weak ties hypothesis, which is first proposed by Granovetter,^8^ is a central concept in the social network analysis.^9,10^ Weak ties not only play a role in effecting social cohesion^11^ but also are helpful for stabilizing complex systems under most conditions.^12^ Recently, weak ties have been shown to destabilize ecosystems under specific conditions.^13^

Epidemic spreading,^14–17^ a fundamental dynamical process, is one of the most important subjects in the complex network theory.^18–22^ Inspired by the significant effects of weak ties on the dynamics on networks,^12^ many recent works have contributed to understanding the interplay between weak ties and spreading dynamics on community networks.^23–27^ Onnela *et al.* found that weak ties can significantly slow the diffusion process, leading to the dynamic trapping of information on communities.^28,29^ As weak ties are removed gradually, the coverage of information will drop sharply.^30^ On adaptive networks, strong communities with weak ties may prevent disease propagation.^31,32^

In order to assess the accuracy and the forecasting capabilities of numerical models, the predictability of outbreaks has been investigated in many studies.^33–37^ Colizza *et al.* studied the effect of airline transportation network on the predictability of epidemic patterns by means of the normalized entropy function^38^ and found that the heterogeneous weight distribution contributes to enhancing the predictability. Crépey *et al.* found that initial conditions such as the degree heterogeneity of the initial seed can induce a large variability on the prediction of the prevalence.^39^ Loecher and Kadtke argued that the random walk centrality (RWC) serves as a better index than degree to predict the prevalence of disease.^40^ Comparing the scale-free network (SFN) with community structure with the random SFN, the predictability of its global prevalence was found to be better.^41^ Considering the relative independence of a local community, Gong *et al.* investigated the prevalence and its variability on the local community, and found that the extraordinarily large variability in the early stage of outbreaks made the prediction of epidemic spreading hard.^42^ In addition, Zhao *et al.* studied how the heterogeneous time delay (HETD) associated with geographical distance influences the spreading speed and the variability of the prevalence. Owing to the correlations between time delay and network hierarchy in the HETD, the epidemic spreading is slowed down obviously and the predictability of the prevalence is reduced remarkably.^43^

On community networks, as mentioned above, weak ties play a very significant role in epidemic dynamics. But until now, there is no study on the effects of weak ties on the predictability of epidemic dynamics. In this paper, we investigate how weak ties influence the predictability of the epidemic dynamics on community networks. We show numerically that both the degree of bridge nodes and the number of weak ties can remarkably influence the predictability of the epidemic spreading on a local community. More importantly, we find out that the variability of the arrival time always displays a complete opposite trend to that of the prevalence, which implies that it is impossible to predict the epidemic spreading in the early stage of outbreaks accurately.

The paper is organized as follows. In Sec. II, we briefly describe the dynamical process on a community network and introduce the quantitative measurements of predictability. In Sec. III, we investigate the effects of the degree of bridge nodes on the predictability of the dynamics. In Sec. IV, the effects of the number of weak ties on the predictability are analyzed. Finally, we draw conclusions in Sec. V.

## II. MODEL INTRODUCTION

### A. The community network with degree heterogeneity

To investigate the effects of weak ties on the predictability of epidemic dynamics, we must first identify which links on community networks are weak ties. Unfortunately, there is not a generally accepted and authenticated community detection algorithm;^18,48,49^ thus, it is difficult to identify weak ties accurately on real-world networks. We here consider a community network model comprised two confined communities *A* and *B*. Except for the community structure, degree heterogeneity is another important feature of real-world community networks.^44–47^ In this study, we focus on the community network with degree heterogeneity. To be specific, two independent Barabási-Albert (BA) scale-free networks^50,51^ with the same size are first produced, and then these two networks are connected by few links. In order to normalize the terms of community network, we define the links between two communities as *weak ties*,^28^ and call the nodes connected by these weak ties *bridge nodes*.^11,52^ Obviously, the network has a strong community structure because of few weak ties. With the increase of the number of weak ties, the community structure will be weakened.

The network modularity *Q*, a popular evaluating indicator in measuring the community structure,^1^ is defined as

$$Q=\sum_{s=1}^{C}\left[\frac{l_{s}}{L}-{\left(\frac{d_{s}}{2L}\right)}^{2}\right],$$ (1)

where $l_{s}$ and $d_{s}$ represent the number of intra-links and the sum of degrees of the nodes in community *s*, respectively; *L* denotes the total link number in the network; and *C* is the number of communities. Here, 0≤Q≤1, the larger *Q* is, the stronger community structure is. However, the *Q* cannot accurately characterize the community strength of a network with two communities.^32^ To address the shortcoming, the normalized $Q_{n}$ is defined as

$$Q_{n}=\frac{Q-Q_{rand}}{Q_{max}-Q_{rand}},$$ (2)

where $Q_{rand}$ corresponds to the random network with the same degree sequence, and $Q_{max}$ is the modularity of the network without inter-community links. After the normalization, $Q_{n}$ is in range [0, 1].

### B. Dynamic process

In many real-world spreading processes, each node's potential infection-activity is limited, and is not strictly equal to its degree.^53^ For epidemic spreading on human contact networks, although a hub individual has many acquaintances, he/she cannot contact all his/her acquaintances once within one time step.^54,55^ Thus, a contact process (CP) model with identical infectivity is proposed to study the epidemic spreading on complex networks,^55–65^ where each infected node can only contact one of its neighbors per unit time. For simplicity, we only study susceptible-infected (SI)^15^ spreading dynamics through numerical simulations, which is a classical model of the CP. In the model, “*S*” and “*I*” represent, respectively, the susceptible (or healthy) and the infected states. At the beginning, a node is selected as the initial infected (i.e., seed) and all other nodes are in “*S*” state. At each time step, each infected node randomly contacts one of its neighbors, and then the contacted neighboring node will be infected with probability λ if it is in the healthy state, or else it will retain its state. Once an individual is infected, it will keep its state forever. To eliminate the stochastic effect of disease propagation, we set λ=1.

Although other disease models such as susceptible-infected-recovered (SIR) are practically more relevant and realistic, the inclusion of more processes and parameters such as the recovery rate *μ* in SIR model complicates the analysis of the problem considered here. For example, in epidemic spreading with unlimited infectivity, the initial seed with a larger degree will result in a higher prevalence due to the existence of recovery rate in SIR model.^66^ However, the simple SI model can still be adopted to well describe the early dynamics of epidemic outbreaks, such as the acquired immune deficiency syndrome (AIDS), gonorrhea, and syphilis.^15^ Owing to the SI model, the effects of different contact patterns on epidemic spreading can be clearly understood.

### C. Statistical parameter

In view of the relative independence of a local community, we take the dynamics and its variability into account. When a disease emerges on the community network, it is very important for a local community to keep a watchful eye on two statistical parameters: the arrival time and the prevalence of disease, where the arrival time of disease $t_{a}$ is defined as the moment that infectious individual first occurs on the community in each realization, and the prevalence *i*(*t*) is the density of infected individuals at time *t*. In order to investigate the predictability of epidemic dynamics, the variability of the arrival time (the prevalence) is defined as the relative variation of the arrival time (the prevalence) given by^39,42,43^

$$\Delta (t_{a})=\frac{\sqrt{〈t_{a}^{2}〉-{〈t_{a}〉}^{2}}}{〈t_{a}〉},$$ (3)

and

$$\Delta [i(t)]=\frac{\sqrt{〈i{\left(t\right)}^{2}〉-{〈i(t)〉}^{2}}}{〈i(t)〉}.$$ (4)

$\Delta (t_{a})=0(\Delta [i(t)]=0)$ denotes that all independent dynamic realizations are essentially the same, and the arrival time (the prevalence) on the network is deterministic. The larger $\Delta (t_{a})(\Delta [i(t)])$ means the worse predictability that a particular realization is far from average over all independent realizations.

## III. THE EFFECT OF WEAK TIES

### A. The effect of weak tie with different degrees

Because of the degree heterogeneity of the community network, there may be different degrees of bridge nodes, that is, a pair of bridge nodes (i.e., $b_{A}$ and $b_{B}$) connected by a weak tie may have different degrees $k_{b}^{A}$ and $k_{b}^{B}$. In the CP, weak ties with the different combinations of $k_{b}^{A}↔k_{b}^{B}$ have different effects on the propagation from the first community *A* to the second community *B*. As a first step towards this, we consider the case of one weak tie between two communities. Reference 42 showed that once the spreading starts on the second community, the seed is irrelevant. That is to say, different $k_{b}^{B}$ hardly affect the epidemic spreading on the second community *B*. Therefore, by varying the $k_{b}^{A}$ only, we investigate how different $k_{b}^{A}$ influence the predictability of the epidemic dynamics on the second community *B*. In simulations, a node with the fixed degree $k_{b}^{A}$ in the first community *A* is connected with a randomly chosen node in the second community *B*.

**FIG. 1.**
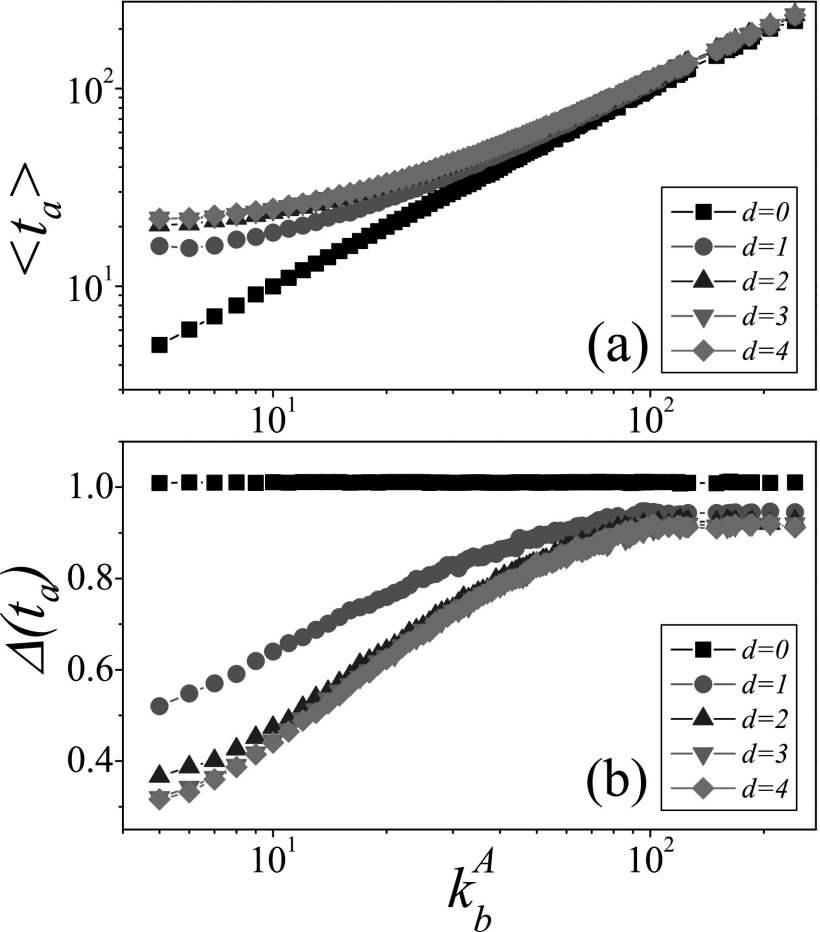
The mean arrival time $〈t_{a}〉$ and its variability $\Delta (t_{a})$ as a function of the degree of the bridge node $k_{b}^{A}$ where the “squares,” “circles,” “triangleups,” “triangledowns,” and “diamonds” denote the cases of the seeds with *d* = 0, 1, 2, 3, and 4, respectively. (a) $〈t_{a}〉$ versus $k_{b}^{A}$, (b) $\Delta (t_{a})$ versus $k_{b}^{A}$. The parameters are chosen as $N_{A}=N_{B}=0.5\times 10^{4},〈k〉=10,\;\text{and}\;\lambda =1$. We perform the experiments on $10^{3}$ different networks, each of which are tested in $10^{3}$ independent realizations.

Fig. 1 shows the mean arrival time $〈t_{a}〉$ and its variability $\Delta (t_{a})$ on the second community *B* when different degrees of bridge nodes are created. When the bridge node $b^{A}$ of the first community *A* is chosen as the seed, that is *d* = 0 (*d* is the geodesic distance from the initial infected node to the bridge node $b^{A}$), the mean arrival time $〈t_{a}〉$ is linear with the degree of the bridge node $k_{b}^{A}$. As the bridge node of the second community $b^{B}$ is one neighbor of the bridge node $b^{A}$, it will be infected with probability $1/k_{b}^{A}$ at each time step, which is obviously a Poisson process. So the mean arrival time $〈t_{a}〉$ is equal to $k_{b}^{A}$ and its relative variation is $\sigma (t_{a})/〈t_{a}〉≃1$. When the disease seed is a node with one step to the bridge node $b^{A}$ (i.e., *d* = 1), the mean arrival time $〈t_{a}〉$ for different $k_{b}^{A}$ will increase compared with the case of *d* = 0. For a small $k_{b}^{A},\;〈t_{a}〉$ is significantly greater than that of *d* = 0, e.g., $〈t_{a}〉(d=1)\approx 15\gg 〈t_{a}〉(d=0)\approx 5$ for $k_{b}^{A}=5$. But for a large $k_{b}^{A}$, the relative change of $〈t_{a}〉$ is very little, e.g., $〈t_{a}〉(d=1)\approx 248>〈t_{a}〉(d=0)\approx 240$ for $k_{b}^{A}=242$. The reason is that the infection of the bridge node $b^{B}$ can be divided into two processes: The bridge node $b^{A}$ is first infected in $〈t_{1}〉\approx 10$ ($t_{1}$ is the time duration of the first process), e.g., $〈t_{a}〉(d=1)-〈t_{a}〉(d=0)\approx 10$ for $k_{b}^{A}=5$ and $〈t_{a}〉(d=1)-〈t_{a}〉(d=0)\approx 8$ for $k_{b}^{A}=242$, and then the bridge node $b^{B}$ is infected by the bridge node $b^{A}$ in $〈t_{2}〉≃k_{b}^{A}$ ($t_{2}$ is the time duration of the second process). With the further increasing of the distance from the seed to the bridge node $b^{A}$ (such as *d* = 3 and 4), the mean arrival times $〈t_{a}〉$ are nearly the same for the fixed $k_{b}^{A}$. It can be understood that owing to the finite size effect of the network with the average shortest path length 〈L〉≈3.7, the bridge node $b^{A}$ is infected till an overall outbreak emerges on the first community *A*.

In Fig. 1(b), the variabilities of the arrival time $\Delta (t_{a})$ for different $k_{b}^{A}$ are shown. When *d* = 0, $\Delta (t_{a})$ for different $k_{b}^{A}$ are approximately equal to 1 because these infections are Poisson processes. Interestingly, compared with the case of *d* = 0, $\Delta (t_{a})$ for *d* = 1 decreases at each $k_{b}^{A}$, that is to say the further distance to the initial seed can lead to the better predictability of the arrival time. As mentioned above, the infection of the bridge node $b^{B}$ has two processes, and thus its variability $\Delta (t_{a})$ can be written as

$$\Delta (t_{a})=\Delta [t_{1}+t_{2}],$$ (5)

where $t_{1}$ and $t_{2}$ denote the time durations of the first process and the second process, respectively. Substituting it into Eq. (3), we obtain

$$\Delta (t_{a})=\frac{\sqrt{D(t_{1}+t_{2})}}{〈t_{1}+t_{2}〉},$$ (6)

where $D(t_{1}+t_{2})=〈{\left(t_{1}+t_{2}\right)}^{2}〉-{〈t_{1}+t_{2}〉}^{2}$. Considering the independence of these two processes, Eq. (6) is reduced to

$$\Delta (t_{a})=\frac{\sqrt{D(t_{1})+D(t_{2})}}{〈t_{1}〉+〈t_{2}〉},$$ (7)

where $D(t_{1})$ and $D(t_{2})$ are the time variances of the first process and the second process, respectively.

**FIG. 2.**
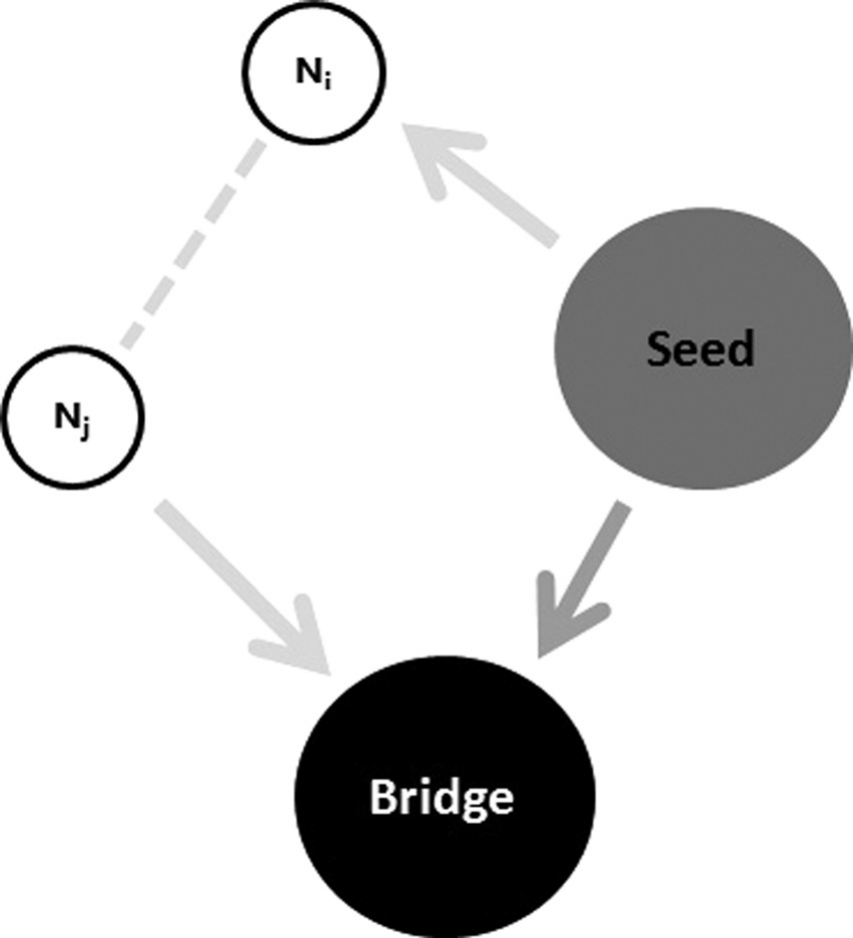
Two spreading pathways through which the bridge node may be infected. The first one is a direct transmission from the seed to the bridge node, and the second one is an indirect transmission from the seed to node *i, j*, and then to the bridge node.

In the first process, there are two basic spreading pathways through which the bridge node $b^{A}$ may be infected. As shown in Fig. 2, the bridge node may be infected directly by the initial seed (i.e., a neighboring node of the bridge node $b^{A}$) with probability $1/k_{s}$; the other route is an indirect transmission of infection from its neighboring nodes except the seed when the overall outbreak occurs on the first community *A*. Although the first route is a Poisson process, the variability $\Delta (t_{1})$ in $t_{1}$ will be less than 1 due to the approximate determinacy of the second pathway. In the second process, the variability $\Delta (t_{2})≃1$ because the infection in $t_{2}$ is a Poisson process. As $D(t)={\left[\Delta (t)〈t〉\right]}^{2}$, we have

$$\Delta (t_{a})=\frac{\sqrt{{\left[\Delta (t_{1})〈t_{1}〉\right]}^{2}+{〈t_{2}〉}^{2}}}{〈t_{1}〉+〈t_{2}〉}.$$ (8)

Obviously, $\Delta (t_{a})$ must be less than 1 when *d* = 1. As $〈t_{2}〉=k_{b}^{A}$ increases with $k_{b}^{A},\;\Delta (t_{a})$ will also increase according to Eq. (8), which is verified by the results in Fig. 1(b). Especially for a very large $k_{b}^{A}$, the variability is very close to 1. It means that although the large degree of the bridge node can delay the mean arrival time of disease, it causes the worst predictability of the arrival time. When $d\geq 2,\;\Delta (t_{1})$ of the first process will be more determined. Thus, $\Delta (t_{a})$ for a small $k_{b}^{A}$ will become small, e.g., $\Delta (t_{a})\approx 0.31(d=4)<\Delta (t_{a})\approx 0.52(d=1)$ for $k_{b}^{A}=5$. The above results demonstrate that when the degree of the bridge node is small, the further distance of the initial seed to the bridge node can result in a better predictability of the arrival time due to the approximate determinacy of the first process, while the variability $\Delta (t_{a})\to 1$ is almost not affected for the very large $k_{b}^{A}$ because of the Poisson property of the second process.

**FIG. 3.**
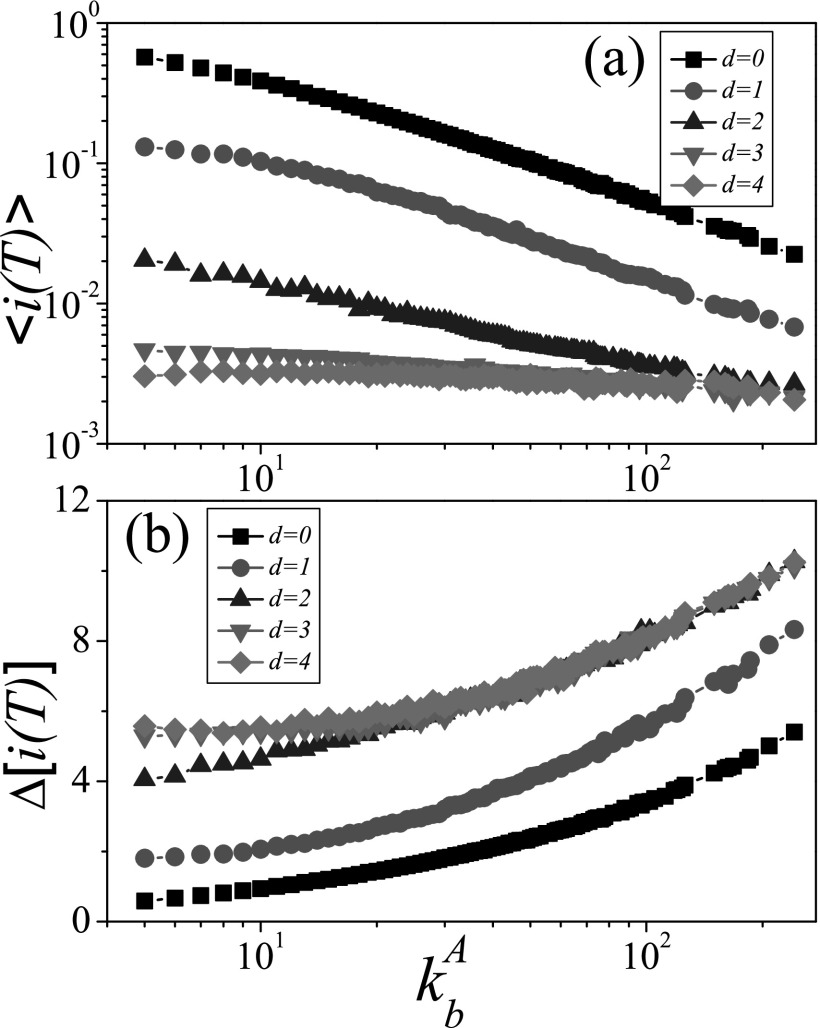
At *T* = 20, the mean prevalence 〈i(T)〉 and its variability Δ[i(T)] as a function of the degree of the bridge node $k_{b}^{A}$ where the “squares,” “circles,” “triangleups,” “triangledowns,” and “diamonds” denote the cases of the seeds with *d* = 0, 1, 2, 3, and 4, respectively. (a) 〈i(T)〉 versus $k_{b}^{A}$, (b) Δ[i(T)] versus $k_{b}^{A}$. The parameters are chosen as $N_{A}=N_{B}=0.5\times 10^{4},〈k〉=10,\;\text{and}\;\lambda =1$. We perform the experiments on $10^{2}$ different networks, each of which are tested in $10^{3}$ independent realizations.

Next, we focus on the statistical parameter Δ[i(t)]. Reference 42 showed that the variability of the prevalence on a local community is very large at the beginning of outbreaks due to the uncertain arrival time of disease, which makes the prediction of the prevalence hard. For this reason, we pay attention to the variability of the prevalence in the early stage of outbreaks. In Fig. 3(a), the mean prevalence 〈i(T)〉 at *T* = 20 decreases with $k_{b}^{A}$ and *d*, which is a complete opposite of the trend of $〈t_{a}〉$ in Fig. 1(a). But Fig. 3(b) shows that its variability increases with $k_{b}^{A}$ and *d*, which is in accordance with the trend of $〈t_{a}〉$. On the one hand, as the very large variability of the prevalence in the early stage is originated from the uncertain arrival time of disease,^42^ it is difficult for the large $k_{b}^{A}$ (corresponding to the large $〈t_{a}〉$) to make sure the arrival of disease within *T* = 20. It will result in the large variability of the prevalence, e.g., Δ[i(T)]≈5.40(d=0) for $k_{b}^{A}=242$. On the other hand, the further distance of the initial seed to the bridge node $b^{A}$ also makes the disease more difficult to arrive at the bridge node $b^{B}$, and can thus cause larger Δ[i(T)], e.g., Δ[i(T)]≈5.60(d=4)≫Δ[i(T)]≈0.60(d=0) for $k_{b}^{A}=5$. The results at other *T* values such as 5, 10, and 30 confirm the conclusion. These results imply that both the large degree of bridge node and the further distance of the initial seed can make the prediction of the prevalence very hard.

### B. The effect of different initial seeds when d = 1

**FIG. 4.**
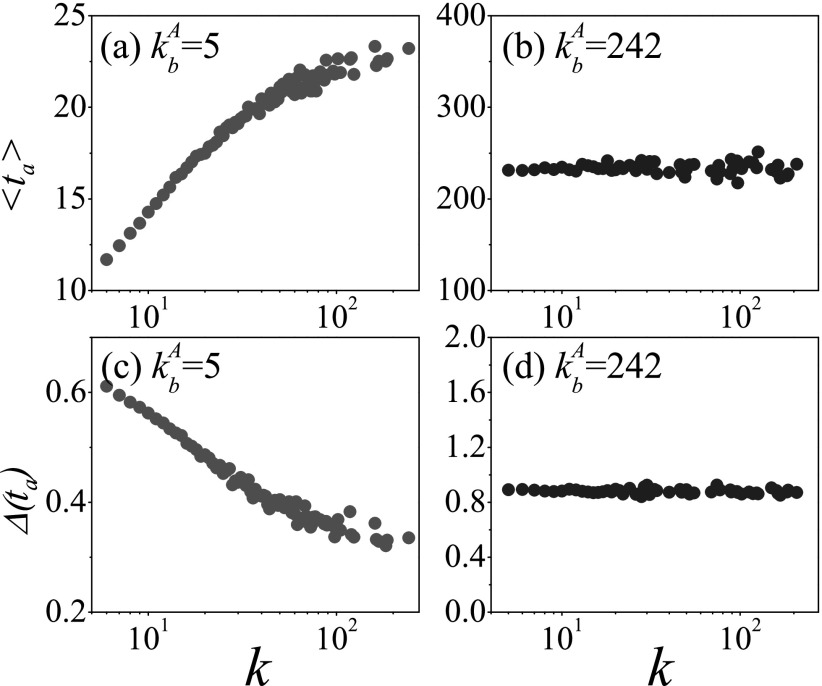
When the distance between the initial seed and the bridge node *d* = 1, the mean arrival time $〈t_{a}〉$ and its variability $\Delta (t_{a})$ as a function of the degree of the initial seed, where $〈t_{a}〉$ versus *k* for $k_{b}^{A}=5$ (a) and $k_{b}^{A}=242$ (b), $\Delta (t_{a})$ versus *k* for $k_{b}^{A}=5$ (c) and $k_{b}^{A}=242$ (d). The results are averaged over $10^{2}\times 10^{3}$ independent realizations on $10^{2}$ networks.

**FIG. 5.**
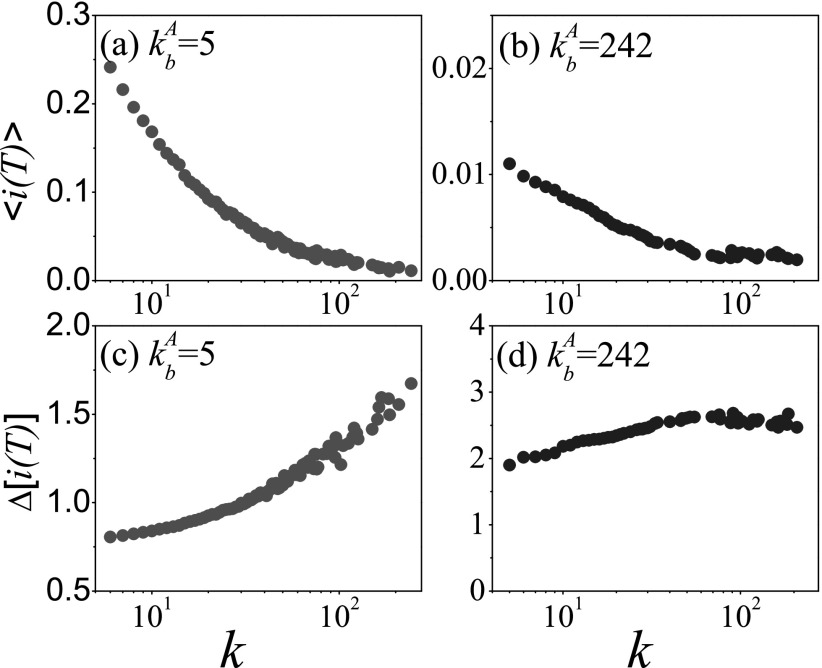
When *d* = 1, the mean prevalence 〈i(T)〉 and its variability Δ[i(T)] at *T* = 20 as a function of the degree of the initial seed, where 〈i(T)〉 versus *k*for $k_{b}^{A}=5$ (a) and $k_{b}^{A}=242$ (b), Δ[i(T)] versus *k* for $k_{b}^{A}=5$ (c) and $k_{b}^{A}=242$ (d). The results are averaged over $10^{2}\times 10^{3}$ independent realizations in $10^{2}$ networks.

From Subsection III A, we know that the degree heterogeneity of the bridge node $b^{A}$ has a significant impact on the predictability of epidemic dynamics. Here, we investigate the effects of different degrees of the initial seed on the variability of the epidemic dynamics on the second community *B* when the distance between the initial seed and the bridge node $b^{A}$ is *d* = 1. As shown in Figs. 4 and 5, when $k_{b}^{A}=5$ is small, $\Delta (t_{a})$ and Δ[i(T)] are obviously affected by the degree of the seed, while there is almost no effect on the variability when $k_{b}^{A}=242$ is large.

When $k_{b}^{A}=5$, the large degree of the initial seed will result in a large $〈t_{a}〉$ (Fig. 4(a)) and a small $\Delta (t_{a})$ (Fig. 4(c)). Owing to the finite contact ability of the initial seed with the large degree, it will cost more time to infect the bridge node $b^{A}$ in the first process, that is the large $〈t_{a}〉$. In the process, the bridge node $b^{A}$ may be infected through two basic pathways. For the initial seed with a small degree, the bridge node $b^{A}$ is infected directly with higher probability. Thus, the first process introduces a larger $\Delta (t_{1})$ because of the randomness of the Poisson process, and thus $\Delta (t_{a})$ in the whole process increases according to Eq. (8). In addition, Fig. 5(c) shows that the variability of the prevalence increases with the degree of the initial seed, which is consistent with the trend of the mean arrival time in Fig. 4(a). We should note that the degree of the initial seed has an opposite effect on the variabilities of the arrival time and the prevalence, which may bring about a great trouble for the pandemic prevention and control.

When $k_{b}^{A}=242$, the whole infection process is dominated by the second Poisson process (i.e., very large $〈t_{2}〉$ in Eq. (8)). Therefore, the different seeds with *d* = 1 cannot affect the variability of epidemic dynamics visibly (see Figs. 4(b), 4(d), 5(b), and 5(d)). However, the very large $\Delta (t_{a})\approx 0.90$ and Δ[i(T)]≈2.50 mean that it is difficult to accurately forecast the epidemic spreading when the degree of the bridge node is large. In order to ensure the universality of the above results, other $k_{b}^{A}$ are also used to simulate the infection process. As expected, all simulations reveal the same conclusion.

## IV. THE EFFECT OF THE NUMBER OF WEAK TIES

In real-world community networks with the modularity Q∈[0.3,0.7],^1^ there are many weak ties between communities. In this section, we would like to understand the effects of the number of weak ties on the predictability of epidemic dynamics. To gain a clear idea of the relation between the modularity *Q* and the number of weak ties, Eq. (1) is expanded as

$$Q=\sum_{s=1}^{2}\left[\frac{l_{s}}{L}-{\left(\frac{d_{s}}{2L}\right)}^{2}\right]=1-\frac{l_{AB}}{L}-{\left(\frac{d_{A}}{2L}\right)}^{2}-{\left(\frac{d_{B}}{2L}\right)}^{2},$$ (9)

where $l_{AB}$ represents the number of weak ties between the community *A* and the community *B*. When the network is connected randomly, $l_{AB}≃(d_{A}d_{B})/2L$, thus $Q_{rand}\to 0$; When $l_{AB}=0$ and $d_{A}=d_{B}$, *Q* reaches the maximum value, i.e., $Q_{max}=0.5$. Therefore, *Q* can only range from 0 to 0.5. Substituting $Q_{rand}=0$ and $Q_{max}=0.5$ into Eq. (2), we have

$$Q_{n}=2Q.$$ (10)

After the standardization, $Q_{n}$ can range from 0 to 1. By adding the number of weak ties $l_{AB}$ between two communities randomly, we can obtain the community networks with different $Q_{n}$.

**FIG. 6.**
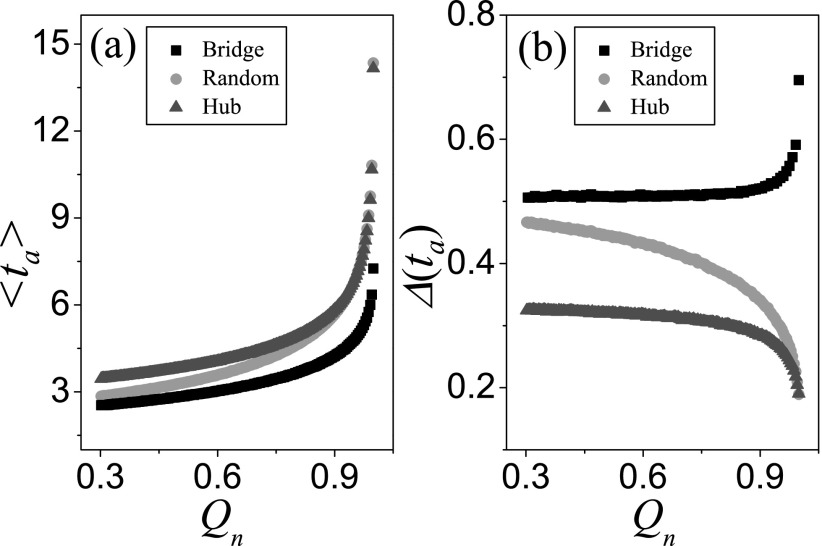
The mean arrival time $〈t_{a}〉$ and its variability $\Delta (t_{a})$ as a function of the modularity $Q_{n}$ where the “squares,” “circles,” and “triangles” denote the cases of the bridge seed, the random seed, and the hub seed, respectively. (a) $〈t_{a}〉$ versus $Q_{n}$, (b) $\Delta (t_{a})$ versus $Q_{n}$. The parameters are chosen as $N_{A}=N_{B}=0.5\times 10^{4},〈k〉=10,\;\text{and}\;\lambda =1$. We perform the experiments on $10^{2}$ different networks, each of which are tested in $10^{3}$ independent realizations.

Fig. 6 shows the case of three kinds of the initial seed: bridge node, random node, and hub. In Fig. 6(a), with the increase of $Q_{n}$, the mean arrival time $〈t_{a}〉$ will increase because fewer weak ties lengthen the distance between two communities. Especially when $Q_{n}\geq 0.9$, the mean arrival time $〈t_{a}〉$ will increase rapidly. Compared with the other two cases, the case of the bridge node chosen as the initial seed has the shortest $〈t_{a}〉$. The case of the random node includes the cases of the bridge node and the non-bridge node, so $〈t_{a}〉$ for the random seed must be longer than that for the bridge seed. For the case of the hub, it has the longest mean arrival time. As the nodes connected by weak ties are chosen randomly, the nodes with a small degree will be more probably chosen as bridge nodes due to the degree heterogeneity, while it is very difficult for the hubs to be bridge nodes. When the degree of the bridge node is small, the initial seed with a large degree leads to the longer $〈t_{a}〉$ (see Fig. 4(a)).

Moreover, the variabilities of the arrival time $\Delta (t_{a})$ for these three cases are compared in Fig. 6(b). As mentioned in Fig. 1(b), the further distance of the initial seed to the bridge node $b^{A}$ can reduce the variability $\Delta (t_{a})$. With the increase of $Q_{n}$, the distance between two communities is lengthened by fewer weak ties, and thus $\Delta (t_{a})$ will decrease. For example, when $Q_{n}$ increases from 0.9 to 1, $\Delta (t_{a})$ for the random case decreases from 0.33 to 0.18 rapidly. For the case of the hub, its arrival time has the most accurate predictability. It is because that the initial seed with a large degree can lead to a low variability when the bridge node $b^{A}$ has a small degree (see Fig. 4(c)). More significant, $\Delta (t_{a})$ for the case of the bridge node increases with the network modularity $Q_{n}$, which is opposite to the other two cases. Even though a bridge node $b_{i}^{A}$ is chosen as the initial seed, the infection to community *B* is not always through the weak tie of the bridge node $b_{i}^{A}$ because of the existence of many other weak ties. That is, there are two optional spreading pathways towards the community *B*: the weak tie of $b_{i}^{A}$ and the other weak ties. Thus, the actual path length of epidemic spreading must be greater than 1. In other words, more links will result in the longer distance of the infection process. Therefore, more weak ties can reduce $\Delta (t_{a})$ due to the increase of the distance between the community *A* and the community *B*. Actually, more weak ties can increase the deterministic of the second optional pathway, and thus enhances the predictability of the arrival time.

**FIG. 7.**
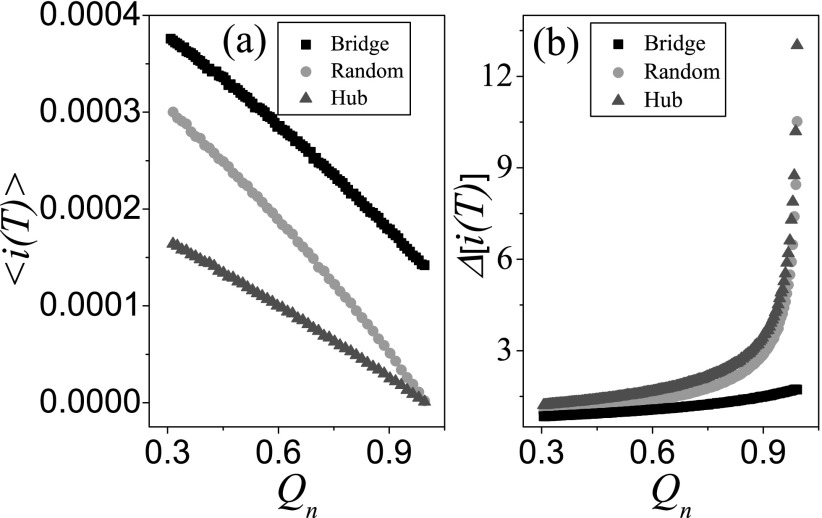
At *T* = 2, the mean prevalence 〈i(T)〉 and its variability Δ[i(T)] as a function of the modularity $Q_{n}$ where the “squares,” “circles,” and “triangles” denote the cases of the bridge seed, the random seed, and the hub seed, respectively. (a) 〈i(T)〉 versus $Q_{n}$, (b) Δ[i(T)] versus $Q_{n}$. We perform the experiments on $10^{2}$ different networks, each of which are tested in $10^{3}$ independent realizations.

Furthermore, the effects of the number of weak ties on the predictability of the prevalence in the early stage of outbreaks are also analyzed in Fig. 7. As the mean arrival time increases with $Q_{n}$ in Fig. 6(a), the prevalence 〈i(T)〉 at *T* = 2 will decrease accordingly in Fig. 7(a). 〈i(T)〉(hub)<〈i(T)〉(random)<〈i(T)〉(bridgenode) is resulted from $〈t_{a}〉(hub)>〈t_{a}〉(random)>〈t_{a}〉(bridgenode)$. Fig. 7(b) shows that the variability of the prevalence Δ[i(T)] increases with $Q_{n}$. For the case of the bridge node, the change of Δ[i(T)] is very little, which means that the bridge node plays a significant role in enhancing the predictability of the prevalence.^42^ For the cases of the randomly chosen node and the hub, Δ[i(T)] increases slowly when $Q_{n}\in [0.3,0.9)$, while Δ[i(T)] increases rapidly when $Q_{n}\in [0.9,1)$ (e.g., Δ[i(T)]≈13.10 for $Q_{n}\approx 0.99$), which is in accordance with the trend of $〈t_{a}〉$. The results at other *T* values (e.g., *T* = 5 and 10) reveal the same conclusion. It implies that the strong community structure can increase the difficulty of the predictability of the prevalence.

## V. CONCLUSIONS

In conclusion, we have studied the effects of weak ties on the predictability of the epidemic dynamics on a heterogeneous network with local community structure. First, we have shown that the degree of bridge nodes can remarkably influence the variabilities of both the arrival time and the prevalence of disease. With the increase of the degree of the bridge node, the mean arrival time and the outbreak (i.e., the prevalence) of disease will be delayed, but their variabilities will still increase because of the Poisson property of the transmission from the bridge node $b^{A}$ to $b^{B}$.

Second, we have shown that the distance between the initial seed and the bridge node, as well as the degree of the initial seed when *d* = 1, has different impacts on the epidemic predictability under different conditions. When the degree of the bridge node is large, the variability of the arrival time is close to 1, and the variability of the prevalence is very large. When the degree of the bridge node is small, the further distance of the initial seed to the bridge node (or the larger degree of the initial seed when *d* = 1) will enhance the predictability of the arrival time due to the approximate determinacy of the indirect transmission in infecting the bridge node $b^{A}$, while the predictability of the prevalence in the early stage will get worse due to the uncertain arrival time of disease. These results suggest that the bridge node with a small degree may be an important detection station for the epidemic control on community networks. Taking global epidemics on the airline network, for example,^38,67^ the slackened precaution at a small international airport may bring a lot of trouble to the prediction and control of disease.

Moreover, we also have analyzed the effects of the number of weak ties on the epidemic predictability where the results of three different initial seeds (i.e., bridge node, random node, and hub) were compared. With the increase of the network modularity, the variabilities of the arrival time for the case of the random node and the hub will decrease slowly when $Q_{n}\in [0.3,0.9]$, and then drop sharply when $Q_{n}$ is greater than 0.9. It is important to note that the variability of the prevalence will increase rapidly when the community strength is strong enough, which is in accordance with the trend of the mean arrival time. By contrast, owing to the Poisson property of the transmission on weak ties, the variability of the arrival time for the case of the bridge node increases with the network modularity, which displays the worst predictability of the arrival time. But the best predictability of the prevalence is observed when the bridge node is first infected, which is originated from the shortest arrival time for this case. These results suggest that although the strong community structure can delay the infection from one community to another, it can also cause the unpredictability of the epidemic spreading on these communities. It is very important to avoid the infection of bridge nodes in the early stage of outbreaks for a better prediction of the spreading on community networks.

The above results provide us further understanding and new perspective in the effect of weak ties on epidemic spreading. This work only focused on the CP model with limited infectivity, but the other type of epidemic spreading (i.e., epidemic spreading with unlimited infectivity) may change the above conclusions qualitatively. Thus, on a heterogeneous network with local community structure, how weak ties influence the predictability of the unlimited spreading is an interesting question.
